# Abiraterone acetate in metastatic castration-resistant prostate cancer – the unanticipated real-world clinical experience

**DOI:** 10.1186/s12894-016-0132-z

**Published:** 2016-03-22

**Authors:** Darren M. C. Poon, Kuen Chan, S. H. Lee, T. W. Chan, Henry Sze, Eric K. C. Lee, Daisy Lam, Michelle F. T. Chan

**Affiliations:** Department of Clinical Oncology, State Key Laboratory in Oncology in South China, Sir YK Pao Centre for Cancer, Hong Kong Cancer Institute and Prince of Wales Hospital, The Chinese University of Hong Kong, Hong Kong, Hong Kong; Department of Clinical Oncology, Pamela Youde Nethersole Eastern Hospital, Hong Kong, Hong Kong; Department of Oncology, Princess Margaret Hospital, Hong Kong, Hong Kong; Department of Clinical Oncology, Queen Elizabeth Hospital, Hong Kong, Hong Kong; Department of Clinical Oncology, Queen Mary Hospital, Hong Kong, Hong Kong; Department of Clinical Oncology, Tuen Mun Hospital, Hong Kong, Hong Kong; Hong Kong Society of Uro-Oncology (HKSUO), Hong Kong, Hong Kong

**Keywords:** Castration-resistant prostate cancer, Abiraterone acetate, Chemo-naïve, Chemotherapy, PSA response

## Abstract

**Background:**

There is much interest in confirming whether the efficacy of abiraterone acetate (AA) demonstrated within the trial setting is reproducible in routine clinical practice. We report the clinical outcome of metastatic castration-resistant prostate cancer (mCRPC) patients treated with AA in real-life clinical practice.

**Methods:**

The clinical records of mCRPC patients treated with AA from all 6 public oncology centers in Hong Kong between August 2011 and December 2014 were reviewed. The treatment efficacy and its determinants, and toxicities were determined.

**Results:**

A total of 110 patients with mCRPC were treated with AA in the review period, of whom 58 were chemo-naive and 52 had received prior chemotherapy (post-chemo). The median follow-up time was 7.5/11.4 months for chemo-naive/post-chemo patients. 6.9/15.4 % of chemo-naive/post-chemo patients had visceral metastases. The median overall survival (OS) and progression-free survival (PFS) were 18.1/15.5 months and 6.7/6.4 months for chemo-naive/post-chemo patients, respectively. Among chemo-naive patients, those with visceral diseases had significantly inferior OS (2.8 vs 18.0 *p* = 0.0007) and PFS (2.8 vs 6.8 months, *p* = 0.0088) than those without. Pain control was comparable in both groups of patients. The most common grade 3 or above toxicities were hypertension (6.9/5.8 %) and hypokalemia (3.4/3.8 %) in chemo-naive/post-chemo patients. In multivariate analysis, the presence of prostate-specific antigen (PSA) response (≥50 % drop of PSA from baseline) within the first 3 months of therapy was associated with favorable OS and PFS in both chemo-naive and post-chemo group.

**Conclusions:**

In clinical practice outside the trial setting, OS after AA in our chemo-naive patient cohort (18.1 months) was considerably shorter than that reported in the COU-AA-302 trial (34.7 months), and the OS was particularly short in those with visceral metastases (2.8 months). Conversely, AA was efficacious in post-chemo patients. AA resulted in comparable pain control in both groups of patients. The presence of PSA response within the first 3 months of treatment was a significant determinant of survival.

## Background

Androgen deprivation therapy (ADT), either medical or surgical, is the backbone of first line treatment for metastatic prostate cancer [1]. While up to 80 % of patients will respond favorably to this therapy; metastatic castration-resistant disease (mCRPC), would be encountered ultimately [2].

Since 2004, docetaxel chemotherapy was the standard of care for patients with mCRPC [3, 4]. More recently, the treatment paradigm had been altered dramatically with the advent of several androgen receptor (AR) pathway targeted agents, new-generation chemotherapy, and immunotherapy [5]. Abiraterone acetate (AA), a potent and irreversible inhibitor of cytochrome-P (CYP)-17 that blocks androgen synthesis, has been shown in large-scale randomized trials to confer significant survival advantage over placebo in both chemo-naïve mCRPC patients and mCRPC patients with prior chemotherapy (post-chemo) [6, 7].

There is much interest in confirming whether the efficacy of AA demonstrated within the trial setting is reproducible in routine clinical practice, in consideration of possible differences in selection of patients, ethnic differences, and other factors in day-to-day practice. In fact, for the case of docetaxel for mCRPC patients, previous retrospective studies had shown unexpectedly higher incidence of febrile neutropenia and less favorable survival outcome compared to that in the trial setting [8, 9]. In the present study, we report on the clinical outcome of AA in patients with mCRPC from all 6 public oncology centers in Hong Kong.

## Methods

### Ethics statement

The study was approved by the institutional review board of the authors’ institutions (Joint Chinese University of Hong Kong – New Territories East Cluster Clinical Research Ethics Committee/Ref no: CRE-2015.481). And permission to access the medical records through the inter-hospital computer network was granted by the aforementioned review board. Furthermore, the principles of the Helsinki Declaration were followed. Informed consent has been exempted by the review board as most of the patients in this study were dead when the data was collected.

### Study population and treatment

In early 2011, AA was approved by the local health authority for use in patients with mCRPC who had received prior chemotherapy, and subsequently in 2012, also for chemo-naïve patients. The present review included mCRPC patients who were started on AA in 6 oncology centers between August 2011 and December 2014. All patients had metastatic prostate cancer which had progressed despite achieving castration-level of testosterone. Enzalutamide, another AR pathway targeted agent, was not accessible during the study period and only be commercially available since October 2015 in our locality [10]. Patients with visceral disease who were medically unfit for, or who declined, chemotherapy, and treated with AA within the period were also included. Patients were treated with 1 g AA once daily in combination with 5 mg prednisone twice a day until disease progression, death or unacceptable toxicity. Clinical and biochemical follow-up with serum prostate-specific antigen (PSA), blood counts, liver and renal profile were regularly undertaken during the treatment period. Serum lactate dehydrogenase (LDH) was not a mandatory parameter to be regularly examined during the treatment. Regular imaging assessment was not mandatory unless clinical suspicion or biochemical progression was evident. Continuation of AA beyond disease progression and post-AA treatments were at the discretion of individual oncologists based on several factors including patient’s preference, medical condition or affordability, physician’s preference and availability of alternative treatment options.

### Data collection and outcomes measures

The electronic clinical records of the patient cohort were retrieved by the inter-hospital computer network. The definition of clinical, biochemical and radiological progressive disease was according to the Prostate Cancer Clinical Trials Working Group (PCWG-2) criteria [11]. Overall survival (OS) and progression-free survival (PFS) were defined as time from first dose of AA to death, and to the first event of clinical, radiographic or PSA progression or death, respectively. Patients who had transient serum PSA level upsurge but not to the extent of biochemical progression (PCWG-2 criteria) followed by a drop, were defined as having PSA flare. PSA doubling time (PSA-DT) was calculated by determining the regression slope of the log PSA against time based on 3 consecutive PSA measurements prior to AA. Patients who had reduction or withdrawal of WHO class II or III analgesics according to the WHO analgesics ladder during or after AA was regarded as having improvement in pain control. Treatment-related toxicities were graded according to the National Cancer Institute Common Terminology Criteria for Adverse Events (CTCAE) 4.02 toxicity scale.

### Statistical analysis

Statistical analysis was performed using the Statistical Package for the Social Sciences (Windows version 17.0.1.80; SPSS Inc, Chicago, US). The updated database as at 1 May 2015 was used for analysis. Kaplan-Meier plots of OS and PFS were obtained for subsets of patients segregated by various potential prognosticators. The log-rank test was employed to assess the difference in outcome between subsets. The variables were also subject to multivariate analyses using the Cox proportional hazards regression model. *P* values ≤ 0.05 were considered significant. The hazard ratio (HR) and the corresponding 95 % confidence interval were calculated.

## Results

### Characteristics of patients

Hundred and ten patients were reviewed, of whom 58 were chemo-naïve and 52 were post-chemo. Table 1 summarizes the characteristics of the patient cohort. The median follow-up duration was 7.5 (range, 1.0–24.6) and 11.43 (range, 1.2–30.2) months for chemo-naïve and post-chemo group respectively. Visceral diseases (non-nodal soft tissue metastases) were present in 4 (6.9 %) chemo-naïve and 8 (15.4 %) post-chemo patients. About 30 % of patients were symptomatic prior the initiation of AA.

**Table 1:** Patient’s characteristics

	Chemo-naïve (*n* = 58)	Post-chemo (*n* = 52)
Age, years		
Median	77	66
Range	56–92	39–85
≥75 (%)	37 (63.8)	11 (21.2)
ECOG performance status, No. (%)		
0–1	36 (62.1)	45 (86.5)
2	18 (31.0)	7 (13.5)
3	4 (6.9)	0
4	0	0
Gleason score at time of initial diagnosis (%)		
<8	24 (41.4)	19 (36.5)
≥8	16 (27.6)	29 (55.8)
Unknown	18 (31.0)	4 (7.7)
Median PSA (range), ug/l	212 (6.22–3095)	191 (4.2–4694)
Median PSA doubling time (range), months	2.1 (0.5–9.0)	2.3 (0.7–13.4)
Symptomatic at presentation^a^, No. (%)	19 (32.8)	16 (30.8)
Baseline Hb (g/dl), median (range)	12 (5.2–15.5)	11.7 (5.7–14.9)
Baseline ALP (IU/l), median (range)	116.5 (40–2960)	119 (45–1857)
Disease location, No. (%)		
Bone only	35 (60.3)	28 (53.8)
Bone	55 (94.8)	50 (96.2)
Lymph node	22 (37.9)	20 (38.5)
Lung	2 (3.4)^b^	3 (5.8)
Liver	3 (5.2)^b^	5 (9.6)
Co-morbidities, No (%)		
Diabetes Mellitus	16 (27.6)	8 (15.4)
Hypertension	32 (55.2)	20 (38.5)
Hyperlipidemia	0	4 (7.7)
Atrial fibrillation	1 (1.7)	1 (1.9)
Congestive heart failure	1 (1.7)	0
No. of previous cytotoxic regimen (%)		
1	0	44 (84.6)
2	0	7 (13.5)
3	0	1 (1.9)
Disease progression prior AA (%)		
Biochemical progression only	42 (72.4)	35 (67.3)
Clinical or radiographic progression	16 (27.6)	17 (32.7)

*Abbreviations: ECOG* Eastern Cooperative Oncology Group, *PSA* prostate specific antigen, *Hb* hemoglobin, *ALP* alkaline phosphatase, *AA* abiraterone acetate

^a^presence of pain prior abiraterone acetate and require WHO level II or above analgesics

^b^One patient with both liver and lung metastases

### Clinical efficacy

#### PSA response

The proportion of patients with PSA response (in about half of patients), PSA flare (about 30 % of patients), and eventual response after PSA flare (about two-thirds of patients with flare) were similar between the chemo-naïve and post-chemo groups (Table 2). All of the PSA response was present within the first 3 months of AA.

**Table 2:** Treatment details

	Chemo-naïve (*n* = 58)	Post-chemo (*n* = 52)
Median duration of AA treatment, month (range)	6.8 (0.6–21.5)	7.1 (0.5–25.0)
PSA response (%)		
≥50 % PSA decline from baseline	36 (62.1)	26 (50.0)
≥90 % PSA decline from baseline	16 (27.6)	8 (15.4)
Median time to PSA nadir, month (range)	3.1 (0.9–15.0)	2.8 (0.5–15.3)
PSA flare (%)		
No. of patients	17 (29.3)	15 (28.8)
Presence of eventual PSA response (≥50 % PSA decline from baseline)	12 (70.6)	10 (66.7)
Pain alleviation during or after AA^a^ (%)	11 (57.9 %)	11 (68.8 %)
Reasons of discontinuing AA (%)		
Disease progression	24 (41.4)	36 (69.2)
Treatment-related complication	3 (5.2)	1 (1.9)
Patient’s decision	3 (5.2)	2 (3.8)
Unknown	1 (1.7)	0
Continuation of AA beyond PD		
No. of patients (%)	13 (22.4)	18 (34.6)
Median time, month (range)	2.8 (1.0–5.8)	2.0 (1.2–16.2)
Subsequent therapy after PD (%)		
Docetaxel	5 (8.6)	2 (3.8)
Cabazitaxel	2 (3.4)	7 (13.5)
Mitoxantrone	0	1 (1.9)
Ketoconazole	0	1 (1.9)

*Abbreviations: PSA* prostate specific antigen, *AA* abiraterone acetate, *PD* disease progression

^a^Withdrawal or reduction of level II or III analgesics according to WHO analgesics ladder

#### Duration of AA treatment and post-AA treatment

The median duration of AA treatment was 6.8 (range, 0.6–21.5) and 7.1 (0.5–25.0) months for chemo-naïve and post-chemo group respectively, with 27 chemo-naïve, and 13 post-chemo patients still under treatment at the time of last follow-up. Disease progression was the major reason of treatment discontinuation (Table 2). Continuation of AA treatment beyond disease progression and post-AA treatments were observed in 13/18 and 7/11 chemo-naïve/post-chemo group respectively.

#### Overall survival and progression-free survival

**Fig. 1:**
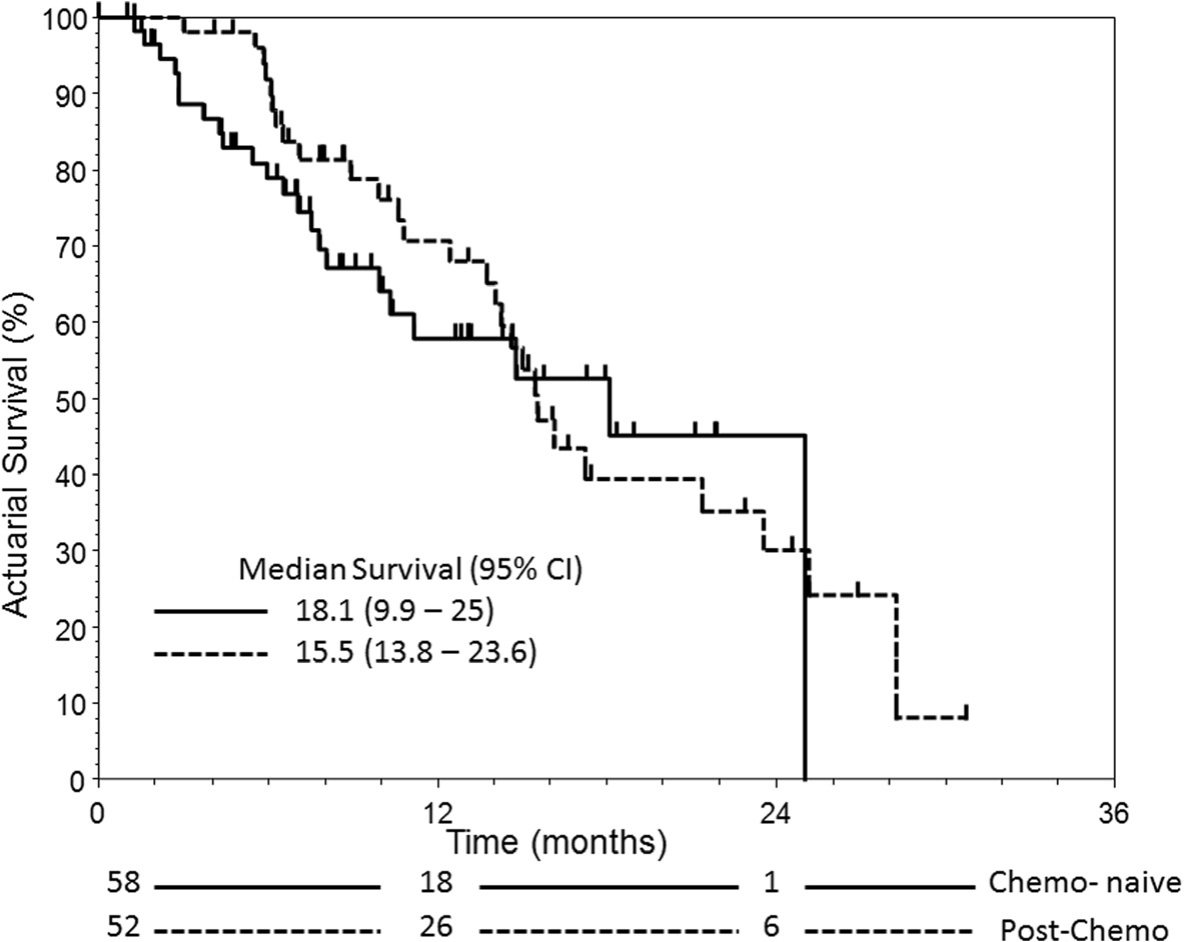
The overall survival for mCRPC patients with (post-chemo) or without prior chemotherapy (chemo-naïve) treated with AA

**Fig. 2:**
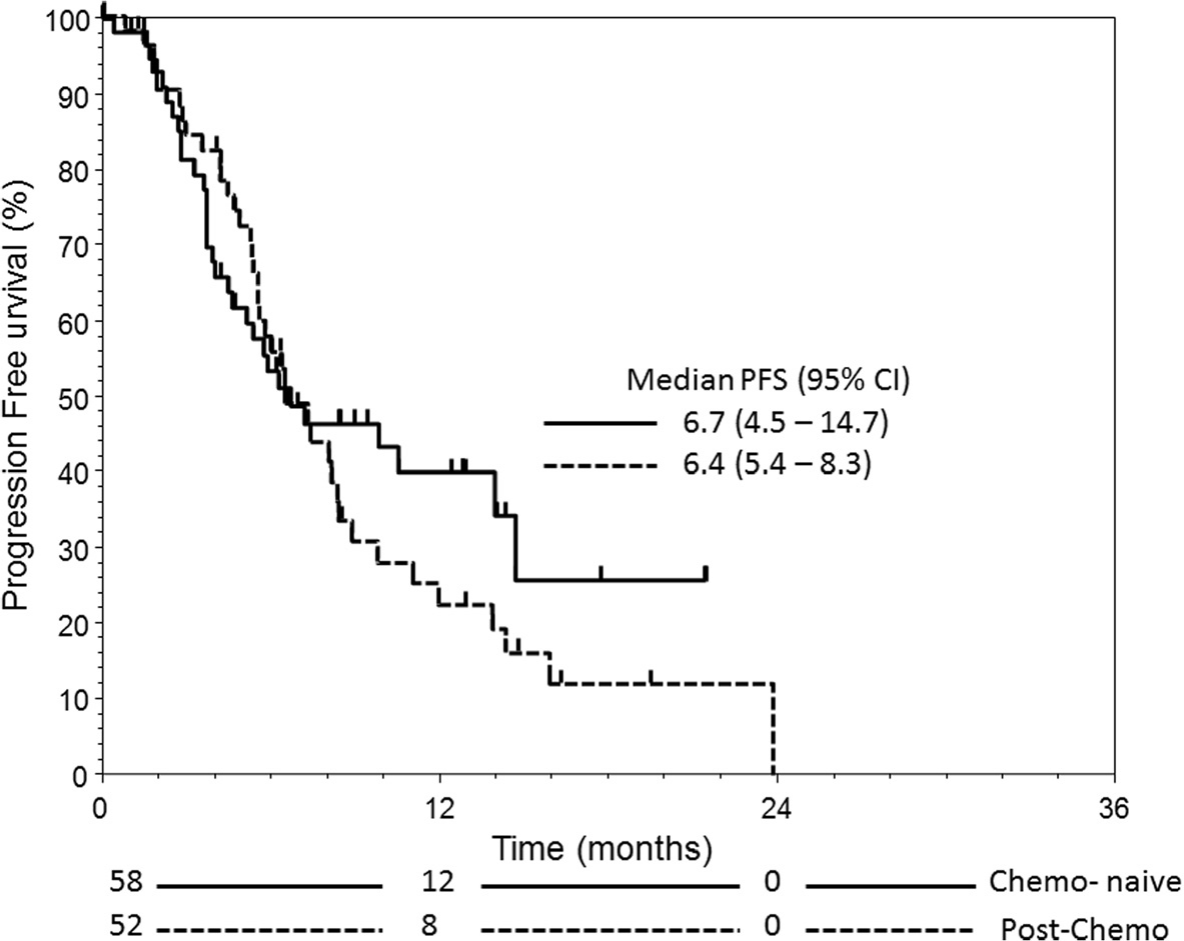
The progression-free survival for mCRPC patients with (post-chemo) or without prior chemotherapy (chemo-naïve) treated with AA

**Fig. 3:**
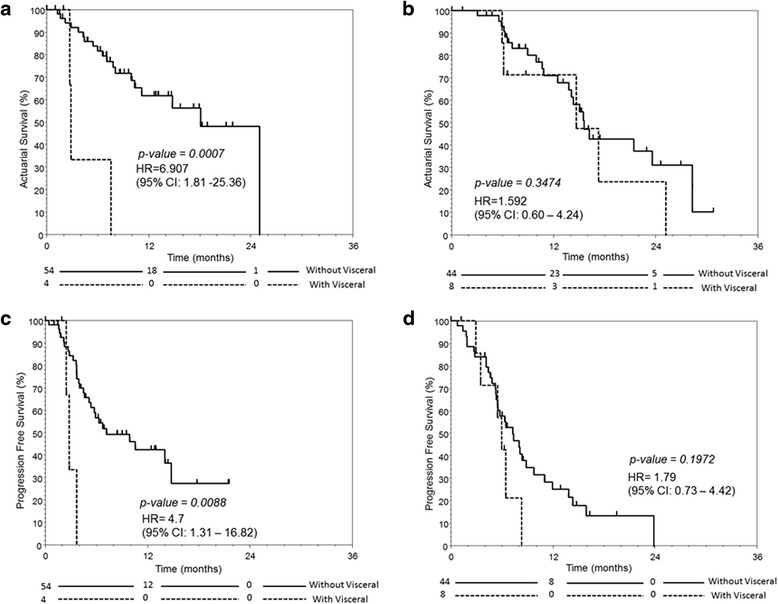
The overall survival for **a** chemo-naïve and **b** post-chemo mCRPC patients with or without visceral disease, and the progression-free survival for **c** chemo-naïve and **d** post-chemo mCRPC patients with or without visceral disease

The median OS was 18.1 (95 % confidence interval (CI): 9.9–25) and 15.5 (95 % CI: 13.8–23.6) months for chemo-naïve and post-chemo group respectively (Fig. 1) whereas their respective median PFS was 6.7 (95 % CI: 4.5–14.7) and 6.4 (95 % CI: 5.4–8.3) months (Fig. 2). Chemo-naïve patients with visceral disease had significantly inferior OS and PFS than those without (OS, 2.8 vs 18.0 months, *p* = 0.0007, HR: 6.907, 95 % CI: 1.81–25.36; PFS, 2.8 vs 6.8 months, *p* = 0.0088, HR: 1.79, 95 % CI: 0.73–4.42). In contrast, the differences in OS and PFS were not significant between patients with or without visceral disease in the post-chemo group (Fig. 3).

#### Pain control

Improvement in pain control was observed in more than half of the patients (Table 2).

### Adverse events

Table 3 shows the treatment-related toxicities in patients treated with AA. In chemo-naïve group, hypokalemia (3.4 %), hypertension (6.9 %) and peripheral edema (5.2 %) were the commonest grade 3 complications, whereas hypertension (5.8 %), hypokalemia (3.8 %) and elevation of liver enzymes (1.9 %) were the commonest grade 3 toxicities in post-chemo group. There was no grade 4 toxicity or treatment-related death among them.

**Table 3:** Adverse events during treatment

	Chemo-naïve (%)				Post-chemo (%)			
	Grade 1	Grade 2	Grade 3	Grade 4	Grade 1	Grade 2	Grade 3	Grade 4
Peripheral edema	6 (10.3)	0	3 (5.2)	0	2 (3.8)	1 (1.9)	0	0
Elevation of liver enzymes	7 (12.1)	0	0	0	3 (5.8)	0	1 (1.9)	0
Hypokalemia	15 (25.9)	1 (1.7)	2 (3.4)	0	9 (17.3)	2 (3.8)	2 (3.8)	0
Hypertension	9 (15.5)	7 (12.1)	4 (6.9)	0	17 (32.7)	3 (5.8)	3 (5.8)	0
Fatigue	1 (1.7)	0	0	0	1 (1.9)	0	0	0
Arthralgia	0	0	0	0	1 (1.9)	0	0	0
Diarrhoea	0	0	0	0	1 (1.9)	0	0	0
Dyspepsia	1 (1.7)	0	0	0	0	0	0	0

### Univariate and multivariate analysis

#### Chemo-naïve group

In univariate analysis, 6 variables, including the presence of visceral disease (HR 6.907, 95 % CI 1.881–25.357, *p* = 0.0007), were significantly determinants of the OS (Table 4). In multivariate analysis, presence of visceral disease (HR 4.8, 95 % CI 1.026–22.465, *p* = 0.0015), presence of PSA response (HR 0.104, 95 % CI 0.025–0.387, *p* = 0.0001), short (<10 months) response to prior ADT (HR 2.656, 95 % CI 1.061–6.648, *p* = 0.0336), ECOG 2 or above (HR 4.907, 95 % CI 1.648–14.612, *p* = 0.0001), and low hemoglobin level (HR 2.696, 95 % CI 0.912–7.7971, *p* = 0.0409) were determinants of OS. Presence of PSA response (HR 0.186, 95 % CI 0.079–0.439, *p* < 0.0001) and presence of visceral disease (HR 5.891, 95 % CI 1.43–24.267, *p* = 0.0126) were determinants of PFS.

**Table 4:** Univariate and multivariate analysis of overall survival and progression-free survival – Chemo-naive group

Factors	Univariate analysis						Multivariate analysis					
	Progression-free survival			Overall survival			Progression-free survival			Overall survival		
	*P* value	HR	95 % CI	*P* value	HR	95 % CI	*P* value	HR	95 % CI	*P* value	HR	95 % CI
Time from ADT to CRPC (<10 vs ≥ 10 months)	0.0306	2.191	1.057–4.542	0.0002	4.566	1.913–10.898	0.816	1.104	0.49–2.489	0.0336	2.656	1.061–6.648
ECOG (2–3 vs 0–1)	0.273	1.51	0.718–3.176	0.0034	3.392	1.426–8.071	N.A.	N.A.	N.A.	0.0001	4.907	1.648–14.612
Age (<75 vs ≥75)	0.2687	0.664	0.319–1.381	0.8875	1.068	0.43–2.653	N.A.	N.A.	N.A.	N.A.	N.A.	N.A.
Gleason score ( ≥8 vs <8)	0.9539	1.023	0.471–2.225	0.9549	0.973	0.376–2.515	N.A.	N.A.	N.A.	N.A.	N.A.	N.A.
Visceral met (yes vs no)	0.0088	4.7	1.313–16.82	0.0007	6.907	1.881–25.357	0.0126	5.891	1.43–24.267	0.0015	4.8	1.026–22.465
Symptomatic (yes vs no)	0.6554	1.183	0.565–2.476	0.1193	1.966	0.826–4.682	N.A.	N.A.	N.A.	N.A.	N.A.	N.A.
PSA doubling time (<2 months vs ≥2 months)	0.1667	1.651	0.804–3.393	0.4794	1.573	0.568–3.319	N.A.	N.A.	N.A.	N.A.	N.A.	N.A.
Baseline PSA ( ≥200 vs <200 ug/l )	0.0014	3.26	1.513–7.021	0.0365	2.558	1.028–6.364	0.0686	2.15	0.933–4.954	0.6339	1.313	0.428–4.038
Baseline ALP ( ≥120 vs <120 IU/l)	0.1464	1.69	0.825–3.466	0.0535	2.459	0.987–6.13	N.A.	N.A.	N.A.	N.A.	N.A.	N.A.
Baseline Hb (<12 vs ≥12 g/dl)	0.1618	1.676	0.805–3.488	0.023	2.712	1.109–6.631	N.A.	N.A.	N.A.	0.0409	2.696	0.912–7.7971
PSA response (yes vs no)	<0.0001	0.135	0.061–0.299	0.0001	0.176	0.067–0.459	<0.0001	0.186	0.079–0.439	0.0001	0.104	0.025–0.387
PSA flare (yes vs no)	0.0623	0.471	0.209–1.06	0.0652	0.373	0.125–1.111	N.A.	N.A.	N.A.	N.A.	N.A.	N.A.
Continuation of AA beyond progression (yes vs no)	N.A.	N.A.	N.A.	0.9863	0.992	0.382–2.574	N.A.	N.A.	N.A.	N.A.	N.A.	N.A.
Post-AA treatment (yes vs no)	N.A.	N.A.	N.A.	0.3604	0.51	0.117–2.219	N.A.	N.A.	N.A.	N.A.	N.A.	N.A.

*Abbreviations: HR* hazard ratio, *95 % CI* 95 % confidence interval, *ADT* androgen deprivation treatment, *CRPC* castration-resistant prostate cancer, *ECOG* Eastern Cooperative Oncology Group, *Symptomatic* presence of pain prior abiraterone acetate and require WHO level II or above analgesics, *PSA* prostate-specific antigen, *ALP* alkaline phosphatase, *Hb* haemoglobin, *PSA* response, ≥50 % drop of PSA from baseline, *PSA flare*, PSA upsurge but not to the extent of biochemical progression, *AA* abiraterone acetate, *N.A.* not applicable

#### Post-chemo group

In multivariate analysis, presence of PSA response (HR 0.213, 95 % CI 0.076–0.592, *p* = 0.0001), Gleason score of ≥ 8 (HR 2.658, 95 % CI 1.13–6.251, *p* = 0.0186) and PSA-DT of < 2 months (HR 3.006, 95 % CI 1.278–7.07, *p* = 0.0289) were determinants of OS, while presence of PSA response (HR 0.403, 95 % CI 0.203–0.797, *p* = 0.0007) was a determinant of PFS (Table 5).

**Table 5:** Univariate and multivariate analysis of overall survival and progression-free survival – Post-chemo group

Factors	Univariate analysis						Multivariate analysis					
	Progression-free survival			Overall survival			Progression-free survival			Overall survival		
	*P* value	HR	95 % CI	*P* value	HR	95 % CI	*P* value	HR	95 % CI	*P* value	HR	95 % CI
Time from ADT to CRPC (<10 vs ≥ 10 months)	0.4867	0.788	0.4–1.549	0.0649	0.476	0.213–1.063	N.A.	N.A.	N.A.	N.A.	N.A.	N.A.
ECOG (2–3 vs 0–1)	0.4084	0.648	0.229–1.832	0.7205	1.248	0.371–4.198	N.A.	N.A.	N.A.	N.A.	N.A.	N.A.
Age (<75 vs ≥75)	0.2988	0.649	0.285–1.478	0.0136	0.303	0.103–0.886	N.A.	N.A.	N.A.	0.3685	0.559	0.155–2.008
Gleason score (≥8 vs <8)	0.0528	1.922	0.98–3.77	0.0236	2.559	1.117–5.846	N.A.	N.A.	N.A.	0.0186	2.658	1.13–6.251
Visceral metastasis (yes vs no)	0.1972	1.793	0.727–4.42	0.3474	1.592	0.598–4.237	N.A.	N.A.	N.A.	N.A.	N.A.	N.A.
Symptomatic (yes vs no)	0.7678	1.112	0.549–2.253	0.637	1.227	0.524–2.87	N.A.	N.A.	N.A.	N.A.	N.A.	N.A.
PSA doubling time (<2 months vs ≥2 months)	0.251	1.459	0.762–2.794	0.0319	2.337	1.054–5.18	N.A.	N.A.	N.A.	0.0289	3.006	1.278–7.07
Baseline PSA (≥200 vs <200 ug/l)	0.3603	1.356	0.703–2.615	0.422	1.367	0.636–2.938	N.A.	N.A.	N.A.	N.A.	N.A.	N.A.
Baseline ALP (≥120 vs <120 IU/l)	0.6113	0.848	0.448–1.605	0.7189	1.151	0.536–2.471	N.A.	N.A.	N.A.	N.A.	N.A.	N.A.
Baseline Hb (<12 vs ≥12 g/dl)	0.2146	1.538	0.774–3.056	0.0811	2.143	0.892–5.149	N.A.	N.A.	N.A.	N.A.	N.A.	N.A.
PSA response (yes vs no)	0.0007	0.336	0.174–0.648	0.0001	0.207	0.089–0.493	0.0007	0.403	0.203–0.797	0.0001	0.213	0.076–0.592
PSA flare (yes vs no)	0.0130	0.38	0.172–0.838	0.2531	0.609	0.257–1.438	0.0987	0.505	0.222–1.149	N.A.	N.A.	N.A.
Refractory to prior chemotherapy (yes vs no)	0.3883	1.376	0.663–2.857	0.2234	1.659	0.729–3.773	N.A.	N.A.	N.A.	N.A.	N.A.	N.A.
Continuation of AA beyond progression (yes vs no)	N.A.	N.A.	N.A.	0.064	0.481	0.218–1.060	N.A.	N.A.	N.A.	N.A.	N.A.	N.A.
Post-AA treatment (yes vs no)	N.A.	N.A.	N.A.	0.3224	0.65	0.275–1.535	N.A.	N.A.	N.A.	N.A.	N.A.	N.A.

*Abbreviations: HR* hazard ratio, *95 % CI* 95 % confidence interval, *ADT* androgen deprivation treatment, *CRPC* castration-resistant prostate cancer, *ECOG* Eastern Cooperative Oncology Group, *Symptomatic* presence of pain prior abiraterone acetate and require WHO analgesic class II or III analgesics, *PSA* prostate-specific antigen, *ALP* alkaline phosphatase, *Hb* haemoglobin, *PSA* response, ≥50 % drop of PSA from baseline, *PSA flare* PSA upsurge but not to the extent of biochemical progression, *AA* abiraterone acetate, *N.A.* not applicable

## Discussion

In the current study, we reported the efficacy and toxicity of AA in mCRPC patients from an unselected patient population in a non-trial setting. The inclusion of all AA-treated patients in all public oncology centers during a defined period serves to provide a representative picture of the efficacy of AA in clinical service setting. The clinical efficacy, notably the OS and PFS, and tolerability of AA in our post-chemo patients was similar to that of the COU-AA-301 study (Table 6), thus reproducing the efficacy of AA in the post-chemotherapy setting. However, unexpectedly, the median OS of chemo-naïve patients in our cohort (of 18.1 months) was remarkably much shorter than that reported in COU-AA-302 study (of 34.7 months). It is noted that our chemo-naïve patients with visceral disease, the patient group that was excluded in the COU-AA-302 study, had significantly inferior survival. If this small subset of poor-prognosis patients was excluded, the OS and PFS of the chemo-naïve patients without visceral metastases were still unfavorable, being similar to the whole group. Thus inclusion of patients with visceral disease in our study cannot entirely explicate the unfavorable survival outcome of chemo-naïve patients. It is also unlikely that the infrequency of post-AA treatment contributes to the unfavorable survival, as the subset of patients given post-AA treatment did not have more favorable survival than those without in the multivariate analysis. We postulate that the inferior survival outcome of our chemo naïve patients could be attributable to a relatively high tumor burden in this patient cohort, compared to that in the COU-302 study. This is supported by a higher baseline PSA level (median: 212 ug/l) in our patients as compared to that in the COU-AA-302 study (median: 42 ug/l). Besides, the inclusion of chemo-naïve patients with poor prognostic features in our study could also account for the unsatisfactory survival results. For example, our patient cohort included symptomatic patients (only asymptomatic or mildly symptomatic patients were included in COU-AA-302 study) and patients with ECOG 2 (patients with ECOG 2 or above were excluded in COU-AA-302 study), and a higher proportion of elderly patients: 63.8 % of patients were age above 75 in our cohort, compared to 34 % in COU-AA-302 study.

**Table 6:** Clinical outcome in the present study and the AA pivotal trial

Survival outcome	Present study (Chemo-naïve)	COU-AA-302 study	Present study (Post-chemo)	COU-AA-301 study
Median OS, months	18.1	34.7	15.5	15.8
Median PFS, months	6.7^a^	16.5^b^	6.4^a^	8.5^c^
				5.6^d^
PSA response, %	62.1	62	50.0	29
Pain control, %	57.9^e^	–	68.8^e^	44^f^

*Abbreviations: OS* overall survival, *PFS* progression-free survival, *PSA response* ≥50 % decline of PSA from baseline, *PSA* prostate-specific antigen

^a^Prostate Cancer Clinical Trials Working Group (PCWG-2) definition

^b^Radiographic PFS

^c^Biochemical PFS

^d^Radiographic PFS

^e^Withdrawal or reduction of level II or III analgesics according to WHO analgesics ladder

^f^Reduction of ≥30 % in the brief pain inventory-short form (BPI-SF) worst pain intensity score over the last 24 h observed at two consecutive evaluations 4 weeks apart without any increase in analgesic usage score; only patients experiencing a pain score ≥4 at baseline were included

It is worth noting that despite the somewhat disappointing survival outcome in chemo-naïve patients treated with AA; nearly 60 % of symptomatic patients had pain alleviation. In fact, such a rate of pain control is similar to that in post-chemo patients, and was also comparable to that in the COU-AA-301 study, despite the pain assessment tools were not identical between ours and the pivotal studies (Table 6). To our knowledge, the present study is the first one to report on efficacy of pain control for symptomatic chemo-naïve patients with AA.

While data on efficacy of AA on chemo-naive patients with visceral metastases or symptomatic disease is being awaited, the present study suggests that patients with high tumor burden, visceral metastases and symptomatic disease may have inferior outcome with AA. An exploratory analysis of the visceral disease subgroup in the COU-AA-301 study [6] has demonstrated that the presence of visceral disease is prognostic but not predictive of the response to AA [12]. Nonetheless, there are growing evidences that the efficacies of therapies are different in chemo-naïve patients and post-chemo patients [13]. In contrast, the presence of symptomatic or visceral metastasis did not confer inferior clinical outcome to docetaxel-based chemotherapy, as reflected by the subgroup analysis in the TAX 327 study [14]. With the lack of randomized trial specifically addressing AA efficacy in chemo-naïve patients with visceral or symptomatic disease, the practice of advocating AA in this particular subgroup should be further scrutinized in the context of clinical trial. Indeed, the data in the present study may support a treatment paradigm of offering AA to mCRPC patients with relatively low tumor burden, and chemotherapy for patients with high tumor burden, and visceral disease. Besides, based on the present study’s data, patients with symptomatic disease may also be considered for AA to help pain control, though the survival outcome is less than favorable.

The achievement of PSA response after AA as a favorable prognosticator is in consistency with prior experience based on the data from COU-AA-302 and 301 studies, in which substantial correlation between survival and PSA kinetics was established [15]. Conversely, the absence of PSA response could potentially be used as a biomarker to select patients earlier for alternative or additional treatment in future clinical trial.

In our study, more than half of patients with initial PSA flare had ultimate PSA response to AA and, furthermore, there was no substantial difference in clinical outcome in patients with or without PSA flare. Consequently, in view of the not uncommon occurrence of PSA flare in some patients, PSA response is better determined at least 12 weeks after treatment, as recommended by the PCWG-2 and the premature discontinuation of AA when encountering initial PSA flare is not suggested [11]. In contrast, the practice of further continuation of AA beyond progression is not advised as the meta-analysis in our study has exemplified that there was no additional enhancement of survival with extended AA.

Short duration of response to prior ADT (<10 months) was associated with an unfavorable survival in chemo-naïve patients with AA in this study. Our finding substantiates the other reports that the short response to ADT was associated with poorer efficacy with AR-pathway targeted therapy, in particular AA, in mCRPC patients [16, 17]. And this echoed the statement made in the latest European consensus that short duration of response to ADT could be used to identify patients with increased risk of primary resistance to AR-pathway targeted agents [18].

Limitations existed in the present study, which include the typical shortcomings of retrospective study such as under-reporting of adverse events, incompleteness of data collection and selection bias etc. However, we consider these limitations would not affect the ability to capture the survival outcome of AA in this study. And the inadequate sample size, difference in follow-up protocols and the policy of post-AA treatment among different hospitals were the other weakness of the current study. Of note, unlike prospective study, regular imaging was not mandatory in our study and this could deprive some patients from other life-prolonging treatment earlier before any clinical or biochemical progression existed. Finally, the follow-up time for the chemo-naive group is comparatively inadequate and the inferior outcome in this group may not be the ultimate result. Our group will plan for another follow-up study in the future.

## Conclusions

The present study reported the unanticipated short survival after AA in chemo-naïve patient outside clinical trial setting. The overall survival was particularly short in those with visceral diseases, and further clinical trial for AA in this subgroup of patients is warranted. In contrast, AA was well tolerated and efficacious in mCRPC patients with prior chemotherapy. AA resulted in comparable pain control in both groups of patients. PSA response, in particular present within the first 3 months after AA, could serve as a prognostic biomarker for survival outcome and may have a potential role in selecting patients for additional or alternative treatment earlier in future clinical trial.

### Ethics approval and consent to participate

The study was approved by the institutional review board of the authors’ institutions (Joint Chinese University of Hong Kong – New Territories East Cluster Clinical Research Ethics Committee/Ref no: CRE-2015.481). Informed consent has been exempted by the review board as most of the patients in this study were dead when the data was collected.

### Availability of data and materials

The dataset supporting the conclusion of this article is included within the article.

## Abbreviations

AA: abiraterone acetate
ADT: androgen deprivation therapy
AR: androgen receptor
mCRPC: metastatic castration-resistant prostate cancer
PCWG-2: Prostate Cancer Clinical Trials Working Group
PSA: prostate-specific antigen
PSA-DT: PSA doubling time
